# Adenovirus-Encoding Virus-Associated RNAs Suppress HDGF Gene Expression to Support Efficient Viral Replication

**DOI:** 10.1371/journal.pone.0108627

**Published:** 2014-10-02

**Authors:** Saki Kondo, Kenji Yoshida, Mariko Suzuki, Izumu Saito, Yumi Kanegae

**Affiliations:** 1 Laboratory of Molecular Genetics, Institute of Medical Science, University of Tokyo, Shirokanedai, Minato-ku, Tokyo, Japan; 2 Regenerative and Cellular Medicine Office, Sumitomo Dainippon Pharma Co., Ltd., Minatojima Minamimachi, Chuo-ku, Kobe, Japan; The University of Tokyo, Japan

## Abstract

Non-coding small RNAs are involved in many physiological responses including viral life cycles. Adenovirus-encoding small RNAs, known as virus-associated RNAs (VA RNAs), are transcribed throughout the replication process in the host cells, and their transcript levels depend on the copy numbers of the viral genome. Therefore, VA RNAs are abundant in infected cells after genome replication, i.e. during the late phase of viral infection. Their function during the late phase is the inhibition of interferon-inducible protein kinase R (PKR) activity to prevent antiviral responses; recently, mivaRNAs, the microRNAs processed from VA RNAs, have been reported to inhibit cellular gene expression. Although VA RNA transcription starts during the early phase, little is known about its function. The reason may be because much smaller amount of VA RNAs are transcribed during the early phase than the late phase. In this study, we applied replication-deficient adenovirus vectors (AdVs) and novel AdVs lacking VA RNA genes to analyze the expression changes in cellular genes mediated by VA RNAs using microarray analysis. AdVs are suitable to examine the function of VA RNAs during the early phase, since they constitutively express VA RNAs but do not replicate except in 293 cells. We found that the expression level of hepatoma-derived growth factor (HDGF) significantly decreased in response to the VA RNAs under replication-deficient condition, and this suppression was also observed during the early phase under replication-competent conditions. The suppression was independent of mivaRNA-induced downregulation, suggesting that the function of VA RNAs during the early phase differs from that during the late phase. Notably, overexpression of HDGF inhibited AdV growth. This is the first report to show the function, in part, of VA RNAs during the early phase that may be contribute to efficient viral growth.

## Introduction

It has become increasingly clear over the past decade that non-coding small RNAs play roles in viral life cycles at various ways [1]–[3]. Hepatitis C virus (HCV) is known to utilize host microRNA miR122, which is specifically expressed and highly abundant in the human liver, to support its efficient replication through its direct attachment to the HCV 5′ non-translation region; thus, miR122 is regarded as a therapeutic target for antiviral intervention [4]–[6]. Moreover, more than two hundred small RNAs derived from viruses have been identified. For example, Epstein-Barr virus (EBV) encodes two small RNAs, EBER-1 and EBER-2 [7]–[9], which modulate the interferon-mediated antiviral response [10].

Adenoviruses (Ads) encode two kinds of non-coding small-RNAs, known as virus-associated (VA) RNAs, VAI and VAII, that consist of 157–160 nucleotides (nts). After Ad infection, the transcription of VA RNAs starts at the same time as the E1A gene and lasts until the late phase. Since the transcription level of VA RNAs increases depending on the number of viral genome copies, VA RNAs in Ad-infected cells are abundant during the late phase, and this is one reason why the functional analysis of VA RNAs during the late phase has been investigated much more frequently than during the early phase.

The VA RNA I (VAI), which is expressed at a level of 10^8^ copies per infected cell during the late phase [11], is required to establish efficient translation in virus-infected cells [12], [13]. Moreover, it is well known that VAI inhibits anti-viral double-stranded RNA (dsRNA)-activated protein kinase (PKR). Also, VAI stabilizes ribosome-associated viral mRNAs, which could lead to enhanced levels of protein synthesis [14]. These findings have indicated that VAI plays a role in creating suitable conditions for viral growth, at least during the late phase of infection. Recently, VA RNAs have been reported to be processed to microRNAs (mivaRNAs) via the cellular RNA-interference (RNAi) machinery, and mivaRNAs disturb cellular DNA expressions during the late phase [15]. However, it has not been investigated the function of VA RNA during the early phase, though the expression of VA RNAs starts immediately during the early phase of viral infection.

E1- and E3-deleted adenovirus vectors (AdVs), known as first-generation (FG) AdVs, have widely been used for the transient expression of transgenes in various cell types. FG AdVs lack E1A gene, an essential for viral replication; consequently, they neither express any viral gene product in target cells nor replicate except in 293 cells, which express E1A gene constitutively. However, since VA RNAs are transcribed by RNA polymerase III, their expressions are independent of E1A-mediated transactivation and they are always transcribed from AdV genome in AdV-infected cells. Therefore, FG AdVs are thought to be a suitable tool for the investigation of VA RNA function during the early phase of viral infection, since they express VA RNAs but do not replicate except in 293 cells. Moreover, these AdVs allow us to study the function of VA RNAs during both early and late phase using 293 cells. For this purpose, AdVs lacking VA RNA genes (VA-deleted AdVs) are essential as a control; however, VA-deleted AdVs have been difficult to generate and produce in quantities sufficient for practical use. Recently, we have developed a novel method for the efficient production of VA-deleted AdVs using a site-specific recombinase FLP [16]. A “pre-vector” containing the VA RNA region flanked by a pair of FRT sequences, which are target sequences for FLP recombinase, is constructed according to a commonly used method for the production of FG AdV [17]. This pre-vector, which is obtained at a high titer, is subsequently used to infect a 293 cell line that constitutively expresses humanized-FLPe [18] (293hde12) [19] so that the VA RNA region is removed from replicating viral genome. Since the excision efficiency of FLP in 293hde12 cells is high enough to remove almost all the VA RNA region from the very high number of viral genome copies, this method can be used to generate a high-titer of VA-deleted AdVs efficiently.

Here, we demonstrated the effect of VA RNAs expressed via FG AdVs on cellular gene expression by comparing the expression patterns between VA-deleted AdV- and FG AdV-infected cells using a microarray analysis. We found that VA RNAs expressed from FG AdVs disturbed the cellular gene expressions. Especially, the expression level of HDGF (hepatoma-derived growth factor; ENSG00000143321.14) was significantly decreased under the replication-deficient conditions; notably, HDGF expression started to decrease even during the early phase of infection in the 293 cells. Moreover, the overexpression of the HDGF gene inhibited viral growth in 293 cells, suggesting that the suppression of HDGF gene expression mediated by the VA RNAs was important for viral growth. This is the first report to show the function of VA RNAs during the early phase of infection.

## Materials and Methods

### Cells and AdVs

Human embryo kidney 293 cell line (ATCC) [20], human lung carcinoma A549 cell line (ATCC) [21], and human hepatocellular carcinoma derived HuH-7 cell line (RIKEN BRC) [22]were cultured in Dulbecco's modified Eagle's medium (DMEM) supplemented with 10% fetal calf serum (FCS). 293hde12 cell line [19], which is a 293 cell line possessing the hFLPe gene [18] (an improved version of the FLPe gene [23]), was cultured in DMEM supplemented with 10% FCS plus geneticin (0.75 mg/mL). After infection with AdVs, the cells were maintained in DMEM supplemented with 5% FCS without geneticin. For AraC (cytosine b-D-arabinofuranoside, hydrochloride: Sigma) treatment, the infected cells were maintained in DMEM supplemented with 5% FCS plus AraC (20 µg/mL).

The FG AdVs were prepared using 293 cells, which constitutively express adenoviral E1 genes and support the replication of E1-substituted AdVs. The VA-deleted AdVs except for HDGF- and GFP-expressing AdVs were prepared according to a method using 293U6VA-1 cells that constitutively express both VAI and VAII. HDGF-expressing and GFP-expressing VA-deleted AdVs were generated as described previously [16]. Briefly, an HDGF-expressing and a GFP-expressing unit under the control of the EF1α promoter was inserted into the SwaI cloning site at the authentic E1 substitution region in the pre-vector cosmid pAxdV-4FVF-w, and the pre-vectors were prepared using 293 cells. Subsequently, the pre-vectors were used to infect 293hde12 cells that constitutively express humanized FLPe recombinase [19] to excise the VA RNA region from the replicating viral genome. The VA-deleted AdVs transcribed less than 1% of the VA RNAs, compared with the FG AdVs, as confirmed using real-time PCR [16]. The VA-deleted AdVs and the FG AdVs were titrated using the methods described by Pei et al [24]. Briefly, the copy numbers of a viral genome that was successfully transduced into infected target cells were measured using qPCR (relative virus titer: rVT). This method enabled us to compare the various titers, since the transduction titer is not influenced by the growth rate of the 293 cells, even if an expressed gene product is deleterious to 293 cells.

### Plasmids

The pVA41da plasmid [16] contains a DNA fragment covering all of VAI and VAII from nt position 10576–11034 of adenovirus type 5. The pBluescript SK (-) (Stratagene) was used as a control. The plasmids were transfected using Transfast (Promega). A pxEFGFP plasmid expressing GFP under the control of the EF1α promoter was used as a transfection control. Two days after the transfection of pVA41da plasmid into 293 cells, the cells were harvested and the total RNAs were extracted as described below to measure the HDGF mRNA levels using qPCR.

### Microarray analysis

VA-deleted AdV (Axd12CARedE) and VA-containing FG AdV (AxCAdsRedE) were infected at an MOI (multiplicity of infection) of 0.5 to A549 cells for 24 h. We prepared triplicate samples for each of the conditions, and total RNA isolation was performed using a Qiagen RNeasy kit (Qiagen). A DNA microarray analysis using Affymetrix Gene-Chip technology was performed as described previously [25]–[27]. Briefly, 100 ng of total RNAs were used as a template for cDNA synthesis, and biotin-labeled cRNA was synthesized with a 3′ IVT Express Kit (Affymetrix). After generating the hybridization cocktails, hybridization to the DNA microarray (Genechip; Human Genome U133 Plus 2.0 Array; Affymetrix) [28] and fluorescent labeling were performed. The microarrays were then scanned with a GeneChip; Scanner 3000 7G System (Affymetrix). The data analysis was performed using GCOS software (Affymetrix). Signal detection and quantification were performed using the MAS5 algorithm with default settings. Global normalization was performed so that the average signal intensity of all the probe sets was equal to 100. For the clustering analysis, the signals were normalized and calculated to the individual scores, and the scores were visualized using Spotfire DecisionCite [29]. The analysis of variance among the groups was also performed using Spotfire DecisionCite and normalized data. All the data acquired by the microarray analysis were deposited in the NCBI Gene Expression Omnibus (NO. GSE58605).

### Quantitative real-time PCR

The total RNA of the infected cells was extracted, and the amount of expressed target RNA and 18S-rRNA (correction standard) were quantified using reverse-transcription and real-time PCR (Applied Biosystems Villa7); the ratio of the target RNA to 18S-rRNA was then calculated. To quantify the AdV genome, the infected total cell DNA was prepared from cells using a previously described method [30], [31] or a DNA preparation kit (TaKaRa Bio). Quantitative PCR (qPCR) was performed to detect the AdV genome using a probe for the pIX gene, as described previously [24]. The amount of chromosomal DNA was simultaneously measured to correct the Ct values of the viral genome per cell. The probes were derived from the sequence of the human β-actin gene for HeLa and HuH-7 cell lines. The qPCR reaction was performed according to the manufacturer's protocol: 50°C for 2 min and 95°C for 10 min, followed by 40 cycles of 95°C for 15 sec and 60°C for 1 min (Applied BioSystems).

### Western blot analysis

Two days after transfection, 293 cells were harvested and the total protein was extracted using NP-40 lysis buffer [50 mM Tris-HCl (pH 8.0), 0.15M NaCl, 5 mM EDTA, 1% NP-40]. The lysates were mixed well in a rotator for 2 h at 4°C, centrifuged at 15,000 rpm for 5 min at 4°C, and the supernatants were collected. Western blotting was performed as described previously [32]. The membrane was incubated for 2 h at room temperature in the presence of anti-HDGF mouse monoclonal antibody (Bio Matrix Research, #BMR00572) diluted to 0.3 µg/mL with PBS-Tween, followed by incubation with peroxidase-conjugated goat anti-mouse IgG+IgM (Jackson Immunoresearch, #115-035-068) diluted to 1/10,000 with PBS-Tween. An anti-actin peptide goat polyclonal antibody (Santa Cruz Biotechnology, #sc-1616) diluted to 1/200 was also detected to show equal loading.

## Results

### HDGF gene expression was downregulated in FG-AdV infected cells

**Figure 1:**
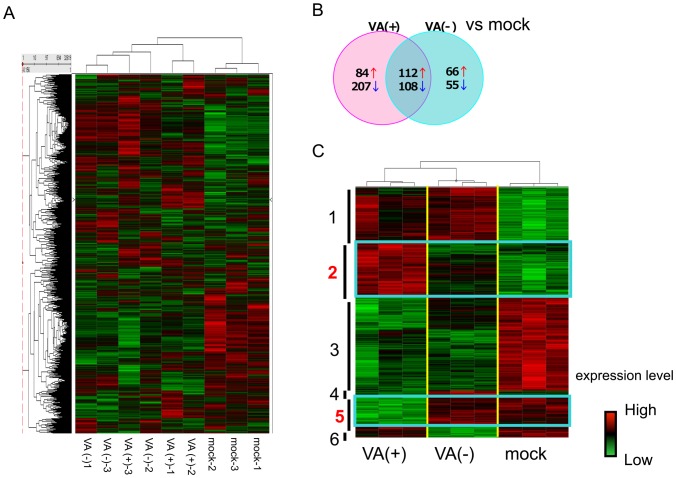
Microarray analysis. Global gene expression analysis of AdV infected A549 cells by Affymetrix microarray. Cells were harvested and total RNA was isolated after 24 h after infection. (A) Hierarchical clustering analysis using 32,619 genes of which expression was determined as “Present” in GCOS software in every sample. (B) The numbers of up or down regulated genes compared with mock infected group (Fold change >1.5, *P*<0.01). Red arrow indicates the numbers of up-regulated genes, and blue arrow indicates the numbers of down-regulated genes. (C) Identification and isolation of VA (+) specific gene clusters by hierarchical clustering analysis. The numbers of target genes were 2,800 genes, which were selected by ANOVA analysis in advance.

To determine whether VA RNAs expressed from FG AdVs disturb cellular gene expression, a microarray analysis was performed. We conducted a hierarchical clustering analysis using data for 32,619 genes, which were determined by GCOS software as being expressed in all the samples. The clusters were divided into two clear groups: namely, a mock group and an AdV-infected group (Figure 1A). Then, we conducted a pairwise comparison and drew a Venn diagram between the mock group vs. VA (−), i.e. VA-deleted AdV, and the mock group vs. VA (+), i.e. FG AdV, according to the criteria of a 1.5-fold change (either an increase or a decrease) and *P*<0.01. In AdV-infected cells, more than 600 genes showed a significant increase/decrease against the mock cells in total. The numbers of VA-(+) specific genes and VA-(−) specific genes were found to be 300 and 100, respectively (Figure 1B). These results indicated that the VA RNAs expressed from AdV do not have a major impact on the expressions of whole genes. Using an ANOVA analysis (*P*<0.01) and a hierarchical clustering analysis, we isolated 6 gene clusters and 2,800 genes that showed different gene expressions between any of the group combinations. Among the 6 clusters, gene clusters 2 and 5 exhibited VA (+)-specific increases in gene expression and VA (+)-specific decreases in gene expression, respectively. According to this gene list (Table S1 in File S1) and literature survey, we finally selected several genes as targets for further research.

Next, we attempted to validate whether our microarray strategy was actually capable of identifying the targets of VA RNAs. We selected a subset of genes, all of which were upregulated or downregulated only after FG AdV infection and not after VA-deleted AdV infection, compared with the mock cells, and measured their transcript levels using quantitative RT-PCR (qPCR) in HeLa cells and HuH-7 cells. The results showed that the expression levels of some of these selected genes were actually changed in response to VA RNAs in both cell lines, except for the PTPRJ gene (Table 1). In contrast, we did not observe any significant changes in the transcript levels of TIA-1 (ENSG00000116001.11), which have been identified as a target for mivaRNA, a microRNA derived from VA RNA [15]. We chose the HDGF gene for further analysis since its transcript was remarkably decreased in both cell lines when FG AdVs were infected.

**Table 1:** Changes in expression levels of cellular genes in response to VA RNAs.

gene	ratio VA(+)/VA(−)	
	HuH-7	HeLa
PAPPA	1.23	1.60
PTPRJ	1.09	1.06
STS1-3	0.82	0.80
HDGF	0.43	0.65
TIA-1	1.10	1.09

Each mRNA level in the HuH-7 cells and HeLa cells was quantified using qPCR, and the ratio of the expression level in FG AdV infected cells (VA (+)) compared with that in VA-deleted AdV infected cells (VA (−)) was calculated.

### HDGF gene expression was suppressed by a lower level of VA RNAs than TIA-1

**Figure 2:**
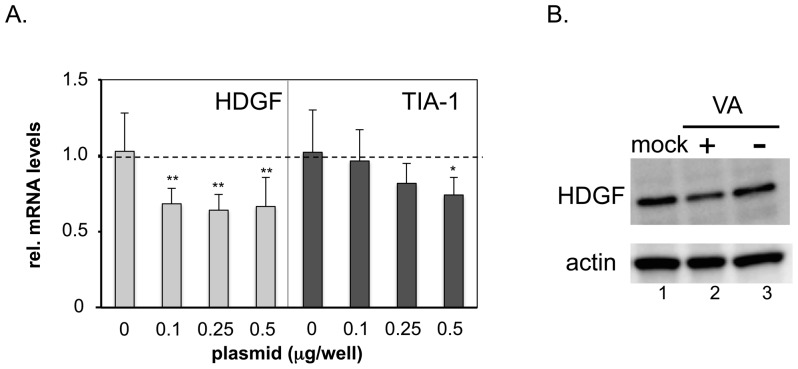
HDGF is downregulated in the presence of VA RNA. (A) HDGF and TIA-1 mRNA levels after VA RNA-expressing plasmid (pVAda41) transfection. RNA (A) and protein (B) were isolated after 48 h from 293 cells transfected with pVA41da or control plasmid. HDGF or TIA-1 and 18S rRNA were quantified using qPCR and plotted for comparison. The expression level in control-plasmid transfected cells was set at 1, and the ratio of the expression levels in all the cases was calculated. The error bars show the standard deviations of three different experiments. *P<0.05, **P<0.01 compared with mock cells (unpaired Student *t*-test). (B) HDGF and actin, used as a loading control, were evaluated using western blot analysis.

To examine whether the VA RNAs expressed from a plasmid also suppress HDGF gene expression, a VA-RNA expressing-plasmid, pVA41da [16], was transfected into 293 cells. Two days later, the total cellular RNA and protein were collected and HDGF expression was measured at the transcript level using qPCR (Figure 2A) and at the protein level using a western blot analysis (Figure 2B), respectively. The result showed that HDGF mRNA was significantly decreased, even in cells with a low level of VA-RNA transduction (Figure 2A, HDGF, 0.1 µg/well), in comparison with control plasmid-transduced cells (Figure 2A, HDGF, 0). In contrast, no significant change in TIA-1 expression was observed in the low VA RNA transduced cells (Figure 2A, TIA-1, 0.1 and 0.25), and it was suppressed only in the highest VA RNA-transduced cells (Figure 2A, TIA-1, 0.5). HDGF suppression mediated by VA RNA was also detected at the protein level (Figure 2B). The HDGF protein was significantly decreased in cells that had been transfected with the VA RNA-expressing plasmid (Figure 2B, lane 2, VA (+)), compared with the mock cells (lane 1, mock) or the control plasmid-transduced cells (lane 3, VA (−)). The suppression of HDGF transcript was also observed in VA RNA-expressing 293 cell lines named 293VA1 and 293VA42 [33], compared with that in the parent 293 cells (Table S2 in File S1). Therefore, VA RNAs suppressed HDGF expression under the conditions other than viral infection, and a smaller amount of VA RNA than TIA-1 was sufficient to suppress HDGF.

### HDGF gene expression was suppressed during the early phase of viral infection

**Figure 3:**
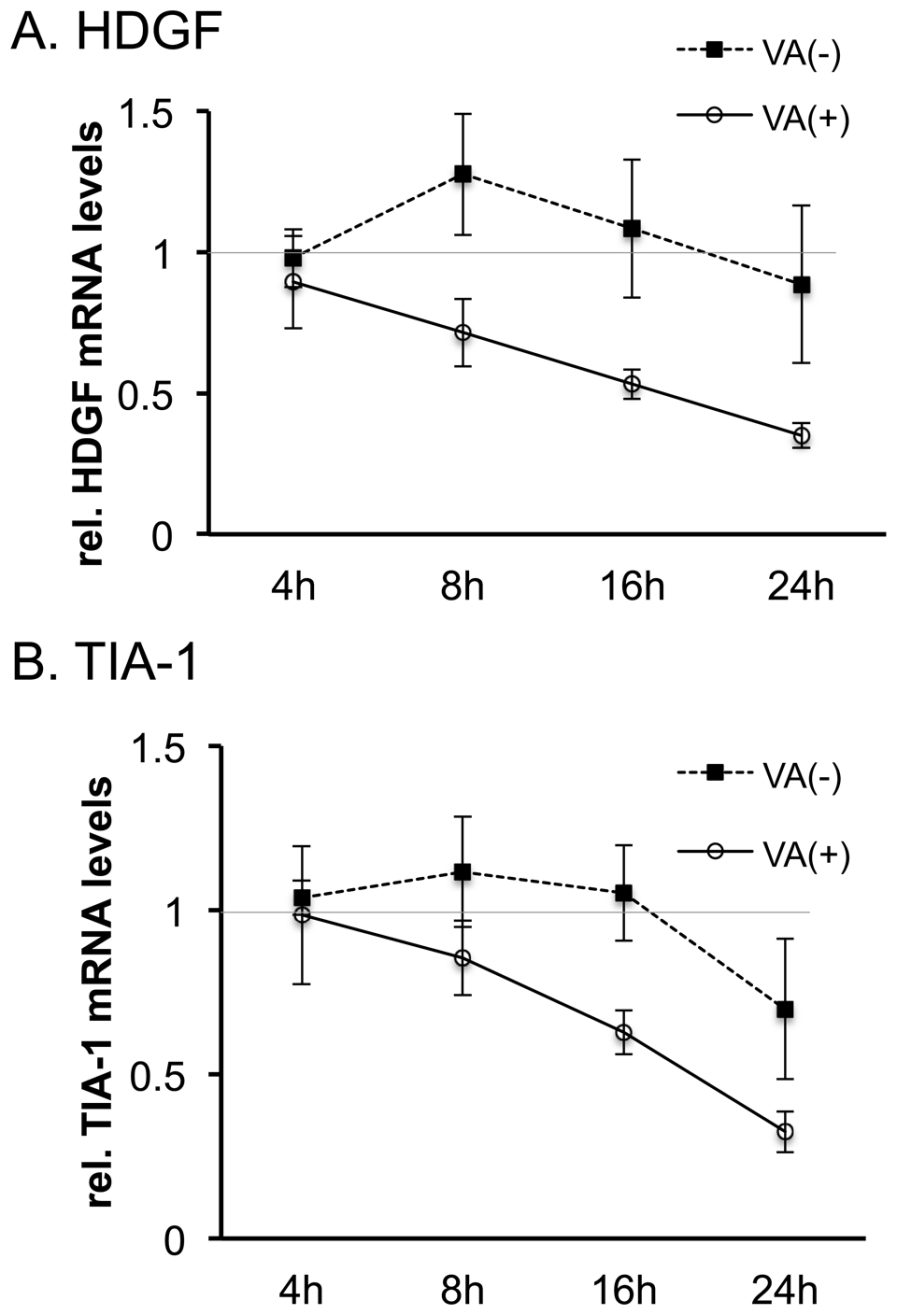
Suppression of HDGF begins during the early phase of viral infection. RNA was isolated from 293 cells infected with VA-deleted AdV (VA (−)) or FG AdV (VA (+)) after the indicated time periods. HDGF (A) and TIA-1 (B) mRNAs were quantified using qPCR. The expression level in uninfected cells was set at 1, and the ratio of the expression level in all the cases was calculated. The error bars show the standard deviations of three different experiments.

To determine the period during which HDGF was downregulated in the adenovirus life cycle, the VA-deleted AdVs and the FG-AdVs were used to infect 293 cells at an MOI of 5. Then, the cellular RNA was isolated to measure the HDGF transcript levels using qPCR at the indicated time points (Figure 3). The VA-deleted AdVs and the FG-AdVs are structurally identical except for their VA RNA expression, and these E1-deleted vectors are able to replicate in 293 cells because the E1 proteins are supplied *in trans*. The results showed that the transcript levels of HDGF started to decrease at 8 h after infection (early phase) in FG AdV-infected cells (Figure 3A, white circle). Interestingly, after VA-deleted AdV infection, the HDGF level clearly increased above the basal level at 8 h (Figure 3A, black square). This induction of HDGF expression after VA-deleted AdV infection was also observed under replication-deficient conditions in HuH-7 cells (Figure S1 in File S1, bars 1 and 3). In contrast, the TIA-1 mRNA level was similar to the basal level at 8 h and it obviously decreased to comparable level with HDGF only at 16 h (late phase) after FG-AdV infection (Figure 3B, white circle), whereas no significant upregulation was observed after VA-deleted AdV infection (Figure 3B, black square). Since the replication of the viral genome occurs at around 8 h after infection, these results showed that the suppression of HDGF and TIA-1 began during the early and late phases of viral infection, respectively. The results for TIA-1 suppression using AdVs were consistent with those of a previous report indicating that TIA-1 is downregulated during the late phase of infection with wild-type adenovirus [15]. We further examined the point that the HDGF level increased to more than 125% of the steady-state level at 8 h after VA-deleted AdV infection (Figure 3A), though the TIA-1 level did not (Figure 3B).

### VA RNAs suppressed the upregulation of HDGF gene expression during the early phase of viral infection

**Figure 4:**
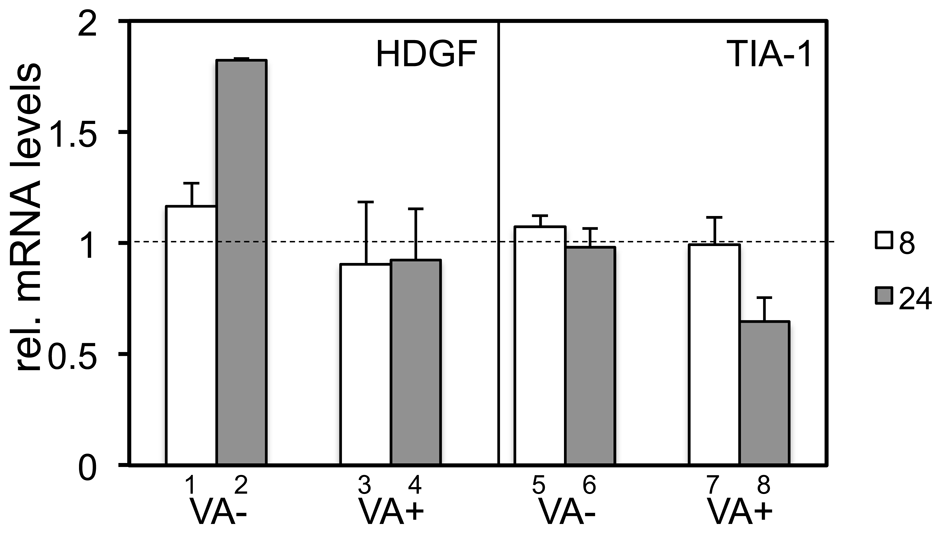
HDGF is upregulated during the early phase of viral infection in VA-deleted AdV-infected 293 cells. RNA was isolated from AraC-treated 293 cells infected with VA-deleted AdV (VA (−)) or FG AdV (VA (+)) after 8 h (white bars) and 24 h (gray bars), and each mRNA level was quantified using qPCR. The expression level in uninfected cells was set at 1, and the ratio of the expression level in all the cases was calculated. The error bars show the standard deviations of three different experiments.

Although the replication of AdV genome starts 8 h after infection, the possibility that the infected cells at 8 h might contain cells reaching late phase cannot be ruled out. To examine the change in HDGF gene expression strictly during the early phase, VA-deleted and FG AdV-infected 293 cells were treated with AraC (cytosine β-D-arabinofuranosde hydrochloride), a nucleoside analog. AraC inhibits viral DNA replication and the transition from the early phase to the late phase; thus, AraC treatment amplifies the effect during the early phase. After the isolation of cellular RNA at 8 h and 24 h after infection, the transcript level of each gene was measured using qPCR and the relative mRNA level of each gene against the steady state level was calculated (Figure 4). The levels of both transcripts in AraC-treated cells at 8 h (white bars) was expected to be similar to those in untreated cells, since the replication of the viral genome had not yet started at this time point regardless of AraC treatment. Remarkably, the induction of HDGF after VA-deleted AdV infection against uninfected cells was detected at much higher levels at 24 h (bar 2) than at 8 h (bar 1). Since AraC amplifies the effect during the early phase, this result confirmed that HDGF gene expression is induced during the early phase of infection. Furthermore, after FG AdV infection with AraC treatment, no significant suppression was observed even at 24 h (bar 4), although without AraC the HDGF level was obviously decreased (Figure 3A, white circle). This result suggested that the increase in HDGF is offset by VA RNAs at 24 h in the presence of AraC, and the amount of VA RNA is not sufficient to decrease this high HDGF level below the basal level. In contrast, no significant change was observed in the TIA-1 level after VA-deleted AdV infection in AraC-treated cells (bars 5 and 6) and at 8 h after FG AdV infection (bar 7), as expected. However, the TIA-1 level was decreased at 24 h after FG AdV infection even in AraC-treated cells (bar 8), though AraC treatment inhibits transition to the late phase. The reason for this observation is unknown, but the amount of accumulated VA RNA might be sufficient for processing to mivaRNAs to suppress TIA-1 expression, which was not increased after AdV infection. Although VA RNAs suppressed both HDGF and TIA-1, the results shown here suggested that the suppression mechanism mediated by VA RNA is different from each other.

### Overexpression of HDGF gene inhibited VA-deleted AdV replication

**Figure 5:**
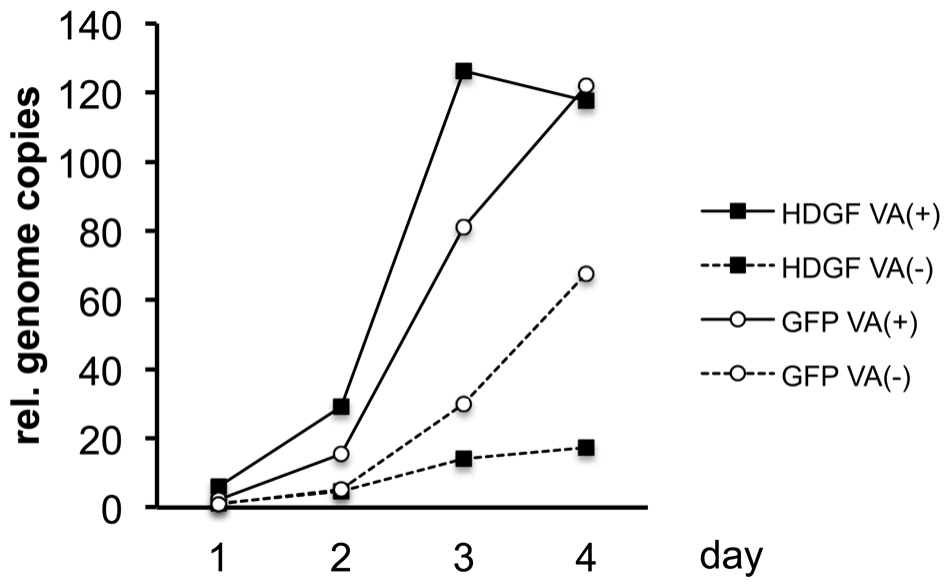
AdV growth in 293 cells. Total DNA was isolated from VA-deleted AdVs (VA (−)) or FG AdVs (VA (+)) infected 293 cells and each AdV genome copy was quantified using qPCR. The level of AdV genome in 293 cells after infection with GFP-expressing FG AdV on day1 was set at 1, and the ratio of the expression level in all the cases was calculated. Three independent experiments were carried out and representative results are shown.

Since the expression of the HDGF gene was increased after VA-deleted AdV infection during the early phase and, therefore, VA RNAs seemed to be responsible for the suppression of the increase in HDGF, we wondered whether HDGF affects viral growth. To test this hypothesis, HDGF-expressing VA-deleted AdVs and FG AdVs were constructed and used to infect 293 cells; the growth efficiency of each AdV was then determined using qPCR to measure the viral genome copy number (Figure 5). We applied an efficient method of generating the VA-deleted AdVs using a site-specific recombinase FLP [16]. A pre-vector, which contains VA RNA genes flanked with a pair of FRTs that are target sequences of FLP, was generated in 293 cells because it behaves as the same as FG AdVs. Subsequently, an obtained pre-vector with a high titer was used to infect 293hde12 cells [19], which are 293 cells expressing the humanized FLPe gene, to excise VA RNA genes out from the replicating AdV genome. For the efficient production of VA-deleted AdVs, a pre-vector was infected five-times more than for FG-AdV production. Under this condition, all cells are infected at once (one-step infection), and the amount of VA RNAs expressed from a pre-vector is sufficient to support the generation of HDGF-expressing VA-deleted AdVs. After AdV infection, the HDGF gene on the AdV genome is expressed exogenously under the control of a potent EF1α promoter. Therefore, the amount of HDGF protein is probably much higher than the endogenous level during AdV replication in 293 cells.

Each vector was used to infect 293 cells at an MOI of 0.5 and the infected cells were collected after 1 to 4 days. GFP-expressing AdV was used as a control. In this infection condition, only a fraction of cells are infected and the uninfected cells are further infected by the newly produced AdVs (multistep infection). Although the growth of HDGF-expressing and GFP-expressing VA-deleted AdVs (dotted lines) was significantly lower than those of FG AdVs (solid lines), this finding was consistent with previous studies indicating a positive role of VA RNAs in viral growth [16], [34]. The results clearly showed that the overexpression of the HDGF gene did not inhibit FG-AdV growth in comparison with control-FG AdV (solid lines). However, the growth of HDGF-expressing VA-deleted AdVs, the genome of which was only amplified to 20 copies on day 4, was much lower than that of GFP-expressing VA-deleted AdVs (dotted lines), which reached 70 copies. These results showed that the overexpression of the HDGF gene inhibited AdV replication as far as HDGF was not suppressed by VA RNAs.

## Discussion

In this study, we demonstrated that adenovirus encoding VA RNAs suppressed HDGF gene expression. This finding revealed, for the first time, a partial role of VA RNAs in the early phase of viral infection.

The suppression of the HDGF level was observed even in cells infected with replication-deficient FG AdVs, which express a much smaller amount of VA RNAs than replicating viruses. The suppression was also detected during the early phase of viral infection in the AdV replication system, i.e., at 8 h after FG AdV infection in 293 cells. In contrast, we confirmed that TIA-1, which is suppressed by VA RNAs during the late phase of viral infection as reported by Aparicio *et al.* [15], was decreased only when VA RNAs were abundant or during the late phase of infection. Although both of these two genes, HDGF and TIA-1, were suppressed in response to VA RNAs, we revealed that the suppression of HDGF required a much smaller amount of VA RNAs than the suppression of TIA-1. This result led us to conclude that VA RNAs probably have different functions during each phase through the regulation of different gene expressions.

According to the adenovirus life cycle, the expression of E1A gene, which is a transactivator for DNA-polymerase II-dependent viral early-gene expression, starts in the immediately during the early phase. The transcription of VA RNAs mediated by DNA polymerase III is independent of E1A-regulated transcription and, therefore, starts almost at the same time as E1A. The amount of VA RNAs during the early phase is much lower than that during the late phase, since it depends on the number of genome copies, which increases to 100,000 copies per cell in the late phase. Actually, the level of VA RNAs during the early phase was about 200-times lower than that during the late phase (Table S3 in File S1). Therefore, the amount of VA RNAs expressed from replication-deficient FG-AdVs is also much smaller than that during the late phase of viral infection. It has been reported that VA RNAs are processed to microRNAs (mivaRNAs) through cellular RNAi machinery and that knockdown of Dicer using siRNA promotes the growth of VA-deleted adenoviruses [35]. However, mivaRNAs suppress TIA-1 expression only during the late phase [15] and have never been detected during the early phase of viral infection [34], [36]. These findings strongly suggest that VA RNAs are processed to microRNAs only when the VA RNAs are abundant. Therefore, the suppression of HDGF gene expression by VA RNAs may not due to mivaRNAs.

In fact, our reporter assay using luciferase suggested that HDGF may not a target for mivaRNAs (Figure S2 in File S1). There is a putative target sequence for a mivaRNA in the 3′ UTR region of the HDGF gene, and we examined whether it is a target sequence or not. As a result, no significant reduction in luciferase activity was detected when the putative seed sequence was cloned into the downstream of luciferase gene. The result suggested that, at least, the known mivaRNAs are not responsible for HDGF suppression. Together with the results shown in Figure 2 and 4, this finding indicated that the role of VA RNAs during the early phase differs from that during the late phase of viral infection, although further investigation is required to reveal the HDGF suppression mechanism mediated by VA RNAs.

The fact that the amount of VA RNA required for HDGF suppression differs from that required for TIA-1 suppression may explain why our microarray analysis did not detect the TIA-1 gene as a positive target. Aparicio *et al.* used cells transfected with a VA RNA-expressing plasmid for their microarray analysis [15]; however, it is difficult to introduce the same number of plasmid copies into 100% of the cells uniformly. Therefore, the results for cells with a high-copy number of plasmids, rather than those for cells with a low-copy number of plasmids, might be favored if a cell mixture containing both high-copy and low-copy number of plasmids is used for the microarray analysis. Consequently, they identified TIA-1 as a target of mivaRNA. In contrast, our microarray using AdVs for VA RNA transduction enabled us to introduce a small amount of VA RNAs into all the cells present in the dish in a uniform manner [18], allowing us to identify novel target genes of VA RNAs during the early phase of infection.

The E1A gene is essential for the adenovirus life cycle and viruses cannot replicate without E1A, such as AdV, which lacks the E1 genes and replicates only in E1-expressing 293 cells. Recently, the interaction of E1A with a cellular factor, CtBP (transcriptional corepressor C-terminal binding protein), has been reported to be required for the efficient E1A-mediated transactivation of early genes [37]. CtBP was initially discovered during screening for cellular factors binding to, and modulating the activity of E1A protein in Ras-mediated tumorigenesis [38]. CtBP was subsequently shown to play an important role in the regulation of cellular genes involved in growth and differentiation [39]. The C-terminal region of E1A interacts with CtBP, and an adenovirus containing the E1A mutation within the CtBP-binding motif, PLDLS, has been shown to decrease the level of early gene expression and, consequently, to inhibit viral growth.

HDGF has also been reported to be a CtBP-binding protein. Yang and Everett showed that HDGF functions as a transcriptional repressor of the SET and MYND domain containing 1 (SMYD1) gene through its interaction with CtBP using the same binding site as E1A [40]. HDGF is a transcription factor consisting of a nuclear protein with both mitogenic and angiogenic activity that is highly expressed in the developing heart and vasculature. HDGF contains an N-terminal PWWP domain and a C-terminal NLS signal. HDGF interacts with CtBP through a non-canonical binding motif (PKDLF), which is located within the PWWP domain, and represses target gene expression by binding to the promoter region leading to cell proliferation. Since both the HDGF and E1A proteins utilize the same binding site on the N-terminus of CtBP using PXDLS-like motifs [40], [41], HDGF might compete with E1A to interact with CtBP. In other words, adenovirus may suppress the expression of HDGF, a cellular-CtBP binding protein, using VA RNAs so that E1A acquires an advantage for CtBP binding.

Our results showed that the upregulation of HDGF, compared with the steady-state level, was observed after infection with VA-deleted AdVs both during the early phase of the replicating condition and during replication-deficient conditions. Of note, some of other CtBP-binding proteins were also transcriptionally upregulated under the same conditions resulting in HDGF upregulation, and VA RNA suppressed these gene expressions, although the expression changes in these genes were not as noticeable as that of HDGF (Table S4 in File S1). These findings suggest that VA RNAs selectively suppress the induction in gene expressions, resulting in the expression of CtBP-binding proteins that may play a role in competitive inhibition with the E1A-CtBP interaction. Moreover, VA RNAs suppress the expression of these genes before the replication of the viral genome, i.e., during the early phase, because E1A-CtBP functions during this phase [37]. In other words, VA RNAs may act to prevent one of the host-defense mechanisms that lead to the inhibition of E1A function, which is essential for the initiation of viral replication.

In the case of AdV infection at a low MOI, which is similar to the condition for native viral infection, the growth of HDGF-expressing VA-deleted AdVs was much lower than that for GFP-expressing VA-deleted AdVs as well as FG AdVs (Figure 5). This result indicates that HDGF expression inhibits viral growth only when the replication starts from a small amount of virus. Our study using AdV at a low MOI may reflect actual viral infection during the very early phase, since a target cell does not express E1A protein before infection and viral infection does not occur at a high MOI. From this point of view, the suppression of the expression of CtBP-binding proteins mediated by VA RNAs might be advantageous for viral growth.

The E1- and E3-deleted AdVs used in this study are widely applied for various studies including gene therapy. However, this vector has two concerns. One is that it, in fact, expresses viral genes, pIX and VA RNAs. It is known that AdVs cause severe immune responses, and we have reported that a main cause is aberrant expression of immunogenic, viral pIX protein, and the pIX protein is not produced when EF1α promoter is used for transgene expression [42]. In terms of VA RNAs, it has not been clear whether a small amount of VA RNAs transcribed via AdVs affects physiological responses in the infected cells or not. The study described here is the first report to show that the VA RNAs expressed from AdVs disturb cellular gene expressions including a transcription factor, HDGF. Our results strongly suggest that production of VA RNAs would be avoided, if possible, when AdVs are applied for gene therapy, since VA RNAs expressed from FG AdVs may affect various cellular signaling pathways. Disturbance of cellular gene expression caused by VA RNAs might also affect the data in the basic study using AdVs. Moreover, although AdVs are often applied for shRNA expression, VA RNA expressed from AdVs inhibits shRNA activity [33], since VA RNAs utilize cellular RNAi machinery for processing of mivaRNAs. The present study provided further evidence that VA-deleted AdVs are useful and might be substituted for FG AdVs.

## Supporting Information

File S1 **Figure S1**, HDGF is suppressed after FG AdV infection in HuH-7 cells. **Figure S2**, HDGF mRNA is not a direct target of mivaRNAI-138. **Table S1**, Gene list for gene clusters 2 and 5. **Table S2**, HDGF and TIA-1 expression levels in 293 cell lines. **Table S3**, Amount of VA RNAs after FG AdV infection in 293 cells. **Table S4**, Ratio of expression levels of genes known to be CtBP-binding proteins after AdV infection in HuH-7 cells.
(PPT)

## References

[1] Grundhoff A, Sullivan CS (2011) Virus-encoded microRNAs. Virology 411: 325–343. 21277611 10.1016/j.virol.2011.01.002 PMC3052296

[2] Zhou R, Rana TM (2013) RNA-based mechanisms regulating host-virus interactions. Immunol Rev 253: 97–111. 23550641 10.1111/imr.12053 PMC3695692

[3] Narayanan A, Kehn-Hall K, Bailey C, Kashanchi F (2011) Analysis of the roles of HIV-derived microRNAs. Expert Opin Biol Ther 11: 17–29. 21133815 10.1517/14712598.2011.540564

[4] Pedersen IM, Cheng G, Wieland S, Volinia S, Croce CM, et al (2007) Interferon modulation of cellular microRNAs as an antiviral mechanism. Nature 449: 919–922. 17943132 10.1038/nature06205 PMC2748825

[5] Jopling CL, Yi M, Lancaster AM, Lemon SM, Sarnow P (2005) Modulation of hepatitis C virus RNA abundance by a liver-specific MicroRNA. Science 309: 1577–1581. 16141076 10.1126/science.1113329

[6] Fukuhara T, Matsuura Y (2013) Role of miR-122 and lipid metabolism in HCV infection. J Gastroenterol 48: 169–176. 22965312 10.1007/s00535-012-0661-5 PMC3698423

[7] Bornkamm GW (2009) Epstein-Barr virus and its role in the pathogenesis of Burkitt's lymphoma: an unresolved issue. Semin Cancer Biol 19: 351–365. 19619654 10.1016/j.semcancer.2009.07.002

[8] Lerner MR, Andrews NC, Miller G, Steitz JA (1981) Two small RNAs encoded by Epstein-Barr virus and complexed with protein are precipitated by antibodies from patients with systemic lupus erythematosus. Proc Natl Acad Sci U S A 78: 805–809. 6262773 10.1073/pnas.78.2.805 PMC319891

[9] Yajima M, Kanda T, Takada K (2005) Critical role of Epstein-Barr Virus (EBV)-encoded RNA in efficient EBV-induced B-lymphocyte growth transformation. J Virol 79: 4298–4307. 15767430 10.1128/JVI.79.7.4298-4307.2005 PMC1061531

[10] Greifenegger N, Jager M, Kunz-Schughart LA, Wolf H, Schwarzmann F (1998) Epstein-Barr virus small RNA (EBER) genes: differential regulation during lytic viral replication. J Virol 72: 9323–9328. 9765483 10.1128/jvi.72.11.9323-9328.1998 PMC110355

[11] Mathews MB (1995) Structure, function, and evolution of adenovirus virus-associated RNAs. Curr Top Microbiol Immunol 199 Pt 2: 173–187. 7555067 10.1007/978-3-642-79499-5_7

[12] Schneider RJ, Weinberger C, Shenk T (1984) Adenovirus VAI RNA facilitates the initiation of translation in virus-infected cells. Cell 37: 291–298. 6722874 10.1016/0092-8674(84)90325-8

[13] Reichel PA, Merrick WC, Siekierka J, Mathews MB (1985) Regulation of a protein synthesis initiation factor by adenovirus virus-associated RNA. Nature 313: 196–200. 2578613 10.1038/313196a0

[14] O'Malley RP, Duncan RF, Hershey JW, Mathews MB (1989) Modification of protein synthesis initiation factors and the shut-off of host protein synthesis in adenovirus-infected cells. Virology 168: 112–118. 2909985 10.1016/0042-6822(89)90409-1

[15] Aparicio O, Carnero E, Abad X, Razquin N, Guruceaga E, et al (2010) Adenovirus VA RNA-derived miRNAs target cellular genes involved in cell growth, gene expression and DNA repair. Nucleic Acids Res 38: 750–763. 19933264 10.1093/nar/gkp1028 PMC2817457

[16] Maekawa A, Pei Z, Suzuki M, Fukuda H, Ono Y, et al (2013) Efficient production of adenovirus vector lacking genes of virus-associated RNAs that disturb cellular RNAi machinery. Sci Rep 3: 1136. 23355950 10.1038/srep01136 PMC3555086

[17] Fukuda H, Terashima M, Koshikawa M, Kanegae Y, Saito I (2006) Possible mechanism of adenovirus generation from a cloned viral genome tagged with nucleotides at its ends. Microbiol Immunol 50: 643–654. 16924150 10.1111/j.1348-0421.2006.tb03829.x

[18] Kondo S, Takata Y, Nakano M, Saito I, Kanegae Y (2009) Activities of various FLP recombinases expressed by adenovirus vectors in mammalian cells. J Mol Biol 390: 221–230. 19414019 10.1016/j.jmb.2009.04.057

[19] Takata Y, Kondo S, Goda N, Kanegae Y, Saito I (2011) Comparison of efficiency between FLPe and Cre for recombinase-mediated cassette exchange in vitro and in adenovirus vector production. Genes Cells 16: 765–777. 21707874 10.1111/j.1365-2443.2011.01526.x

[20] Graham FL, Smiley J, Russell WC, Nairn R (1977) Characteristics of a human cell line transformed by DNA from human adenovirus type 5. J Gen Virol 36: 59–74. 886304 10.1099/0022-1317-36-1-59

[21] Giard DJ, Aaronson SA, Todaro GJ, Arnstein P, Kersey JH, et al (1973) In vitro cultivation of human tumors: establishment of cell lines derived from a series of solid tumors. J Natl Cancer Inst 51: 1417–1423. 4357758 10.1093/jnci/51.5.1417

[22] Nakabayashi H, Taketa K, Miyano K, Yamane T, Sato J (1982) Growth of human hepatoma cells lines with differentiated functions in chemically defined medium. Cancer Res 42: 3858–3863. 6286115

[23] Buchholz F, Angrand PO, Stewart AF (1998) Improved properties of FLP recombinase evolved by cycling mutagenesis. Nat Biotechnol 16: 657–662. 9661200 10.1038/nbt0798-657

[24] Pei Z, Kondo S, Kanegae Y, Saito I (2012) Copy number of adenoviral vector genome transduced into target cells can be measured using quantitative PCR: application to vector titration. Biochem Biophys Res Commun 417: 945–950. 22202173 10.1016/j.bbrc.2011.12.016

[25] Heishi M, Ichihara J, Teramoto R, Itakura Y, Hayashi K, et al (2006) Global gene expression analysis in liver of obese diabetic db/db mice treated with metformin. Diabetologia 49: 1647–1655. 16752183 10.1007/s00125-006-0271-y

[26] Matsui T, Takano M, Yoshida K, Ono S, Fujisaki C, et al (2012) Neural stem cells directly differentiated from partially reprogrammed fibroblasts rapidly acquire gliogenic competency. Stem Cells 30: 1109–1119. 22467474 10.1002/stem.1091

[27] Ishida N, Hayashi K, Hoshijima M, Ogawa T, Koga S, et al (2002) Large scale gene expression analysis of osteoclastogenesis in vitro and elucidation of NFAT2 as a key regulator. J Biol Chem 277: 41147–41156. 12171919 10.1074/jbc.M205063200

[28] Lockhart DJ, Dong H, Byrne MC, Follettie MT, Gallo MV, et al (1996) Expression monitoring by hybridization to high-density oligonucleotide arrays. Nat Biotechnol 14: 1675–1680. 9634850 10.1038/nbt1296-1675

[29] Kaushal D, Naeve CW (2004) Analyzing and visualizing expression data with Spotfire. Curr Protoc Bioinformatics Chapter 7: Unit 7 9. 10.1002/0471250953.bi0709s7 18428735

[30] Saito I, Groves R, Giulotto E, Rolfe M, Stark GR (1989) Evolution and stability of chromosomal DNA coamplified with the CAD gene. Mol Cell Biol 9: 2445–2452. 2569669 10.1128/mcb.9.6.2445-2452.1989 PMC362317

[31] Nakano M, Odaka K, Takahashi Y, Ishimura M, Saito I, et al (2005) Production of viral vectors using recombinase-mediated cassette exchange. Nucleic Acids Res 33: e76. 15879348 10.1093/nar/gni074 PMC1090444

[32] Baba Y, Nakano M, Yamada Y, Saito I, Kanegae Y (2005) Practical range of effective dose for Cre recombinase-expressing recombinant adenovirus without cell toxicity in mammalian cells. Microbiol Immunol 49: 559–570. 15965304 10.1111/j.1348-0421.2005.tb03753.x

[33] Pei Z, Shi G, Kondo S, Ito M, Maekawa A, et al (2013) Adenovirus vectors lacking virus-associated RNA expression enhance shRNA activity to suppress hepatitis C virus replication. Sci Rep 3: 3575. 24356586 10.1038/srep03575 PMC3868971

[34] Aparicio O, Razquin N, Zaratiegui M, Narvaiza I, Fortes P (2006) Adenovirus virus-associated RNA is processed to functional interfering RNAs involved in virus production. J Virol 80: 1376–1384. 16415015 10.1128/JVI.80.3.1376-1384.2006 PMC1346933

[35] Bennasser Y, Chable-Bessia C, Triboulet R, Gibbings D, Gwizdek C, et al (2011) Competition for XPO5 binding between Dicer mRNA, pre-miRNA and viral RNA regulates human Dicer levels. Nat Struct Mol Biol 18: 323–327. 21297638 10.1038/nsmb.1987 PMC3595992

[36] Andersson MG, Haasnoot PC, Xu N, Berenjian S, Berkhout B, et al (2005) Suppression of RNA interference by adenovirus virus-associated RNA. J Virol 79: 9556–9565. 16014917 10.1128/JVI.79.15.9556-9565.2005 PMC1181602

[37] Subramanian T, Zhao LJ, Chinnadurai G (2013) Interaction of CtBP with adenovirus E1A suppresses immortalization of primary epithelial cells and enhances virus replication during productive infection. Virology 443: 313–320. 23747199 10.1016/j.virol.2013.05.018 PMC3732182

[38] Boyd JM, Subramanian T, Schaeper U, La Regina M, Bayley S, et al (1993) A region in the C-terminus of adenovirus 2/5 E1a protein is required for association with a cellular phosphoprotein and important for the negative modulation of T24-ras mediated transformation, tumorigenesis and metastasis. EMBO J 12: 469–478. 8440238 10.1002/j.1460-2075.1993.tb05679.x PMC413230

[39] Chinnadurai G (2002) CtBP, an unconventional transcriptional corepressor in development and oncogenesis. Mol Cell 9: 213–224. 11864595 10.1016/s1097-2765(02)00443-4

[40] Yang J, Everett AD (2009) Hepatoma-derived growth factor represses SET and MYND domain containing 1 gene expression through interaction with C-terminal binding protein. J Mol Biol 386: 938–950. 19162039 10.1016/j.jmb.2008.12.080 PMC2752746

[41] Turner J, Crossley M (2001) The CtBP family: enigmatic and enzymatic transcriptional co-repressors. Bioessays 23: 683–690. 11494316 10.1002/bies.1097

[42] Nakai M, Komiya K, Murata M, Kimura T, Kanaoka M, et al (2007) Expression of pIX gene induced by transgene promoter: possible cause of host immune response in first-generation adenoviral vectors. Hum Gene Ther 18: 925–936. 17907966 10.1089/hum.2007.085

