# Supplementary material for: Adenovirus-Encoding Virus-Associated RNAs Suppress HDGF Gene Expression to Support Efficient Viral Replication
Source: PLoS One. 2014 Oct 2;9(10):e108627. doi: 10.1371/journal.pone.0108627 (PMC4183520; doi:10.1371/journal.pone.0108627)
Supplement: File S1 — Figure S1, HDGF is suppressed after FG AdV infection in HuH-7 cells. Figure S2, HDGF mRNA is not a direct target of mivaRNAI-138. Table S1, Gene list for gene clusters 2 and 5. Table S2, HDGF and TIA-1 expression levels in 293 cell lines. Table S3, Amount of VA RNAs after FG AdV infection in 293 cells. Table S4, Ratio of expression levels of genes known to be CtBP-binding proteins after AdV infection in HuH-7 cells. (PPT) [file pone.0108627.s001.ppt]

## Slide 1
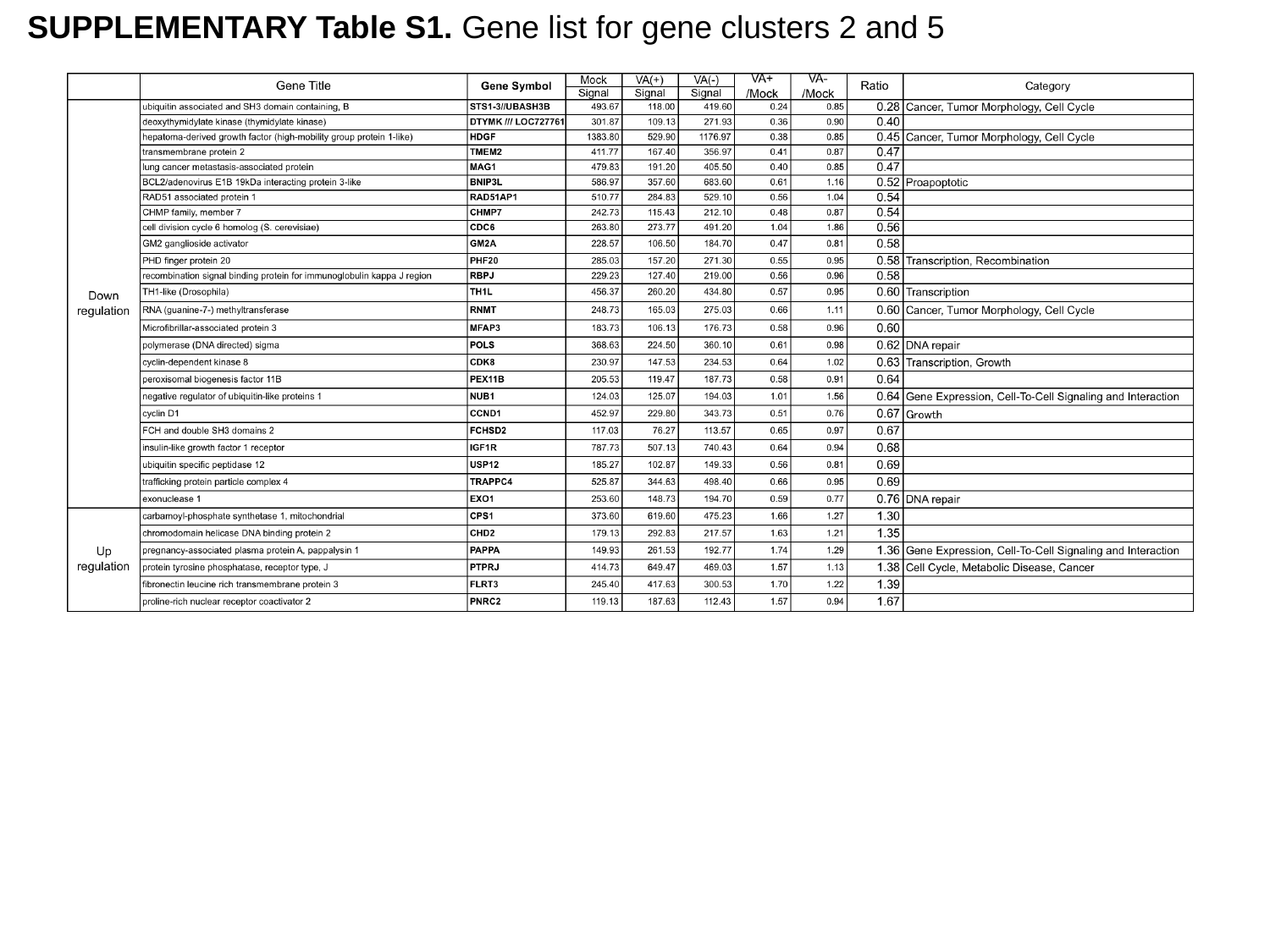

SUPPLEMENTARY Table S1. Gene list for gene clusters 2 and 5

## Slide 2
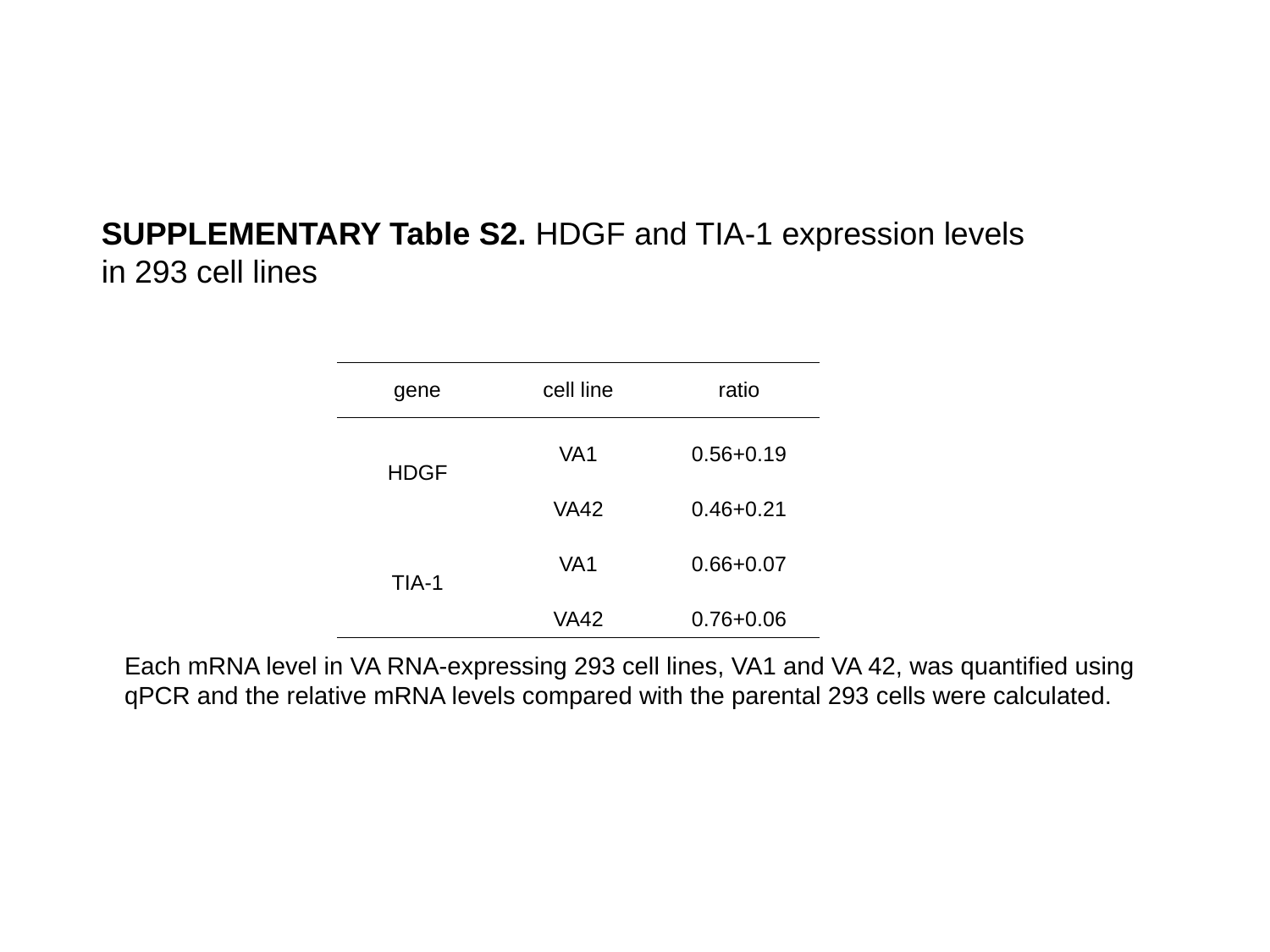

SUPPLEMENTARY Table S2. HDGF and TIA-1 expression levels in 293 cell lines
| | gene | cell line | ratio |
| --- | --- | --- | --- |
| | HDGF | VA1 | 0.56+0.19 |
| | | VA42 | 0.46+0.21 |
| | TIA-1 | VA1 | 0.66+0.07 |
| | | VA42 | 0.76+0.06 |
Each mRNA level in VA RNA-expressing 293 cell lines, VA1 and VA 42, was quantified using qPCR and the relative mRNA levels compared with the parental 293 cells were calculated.

## Slide 3
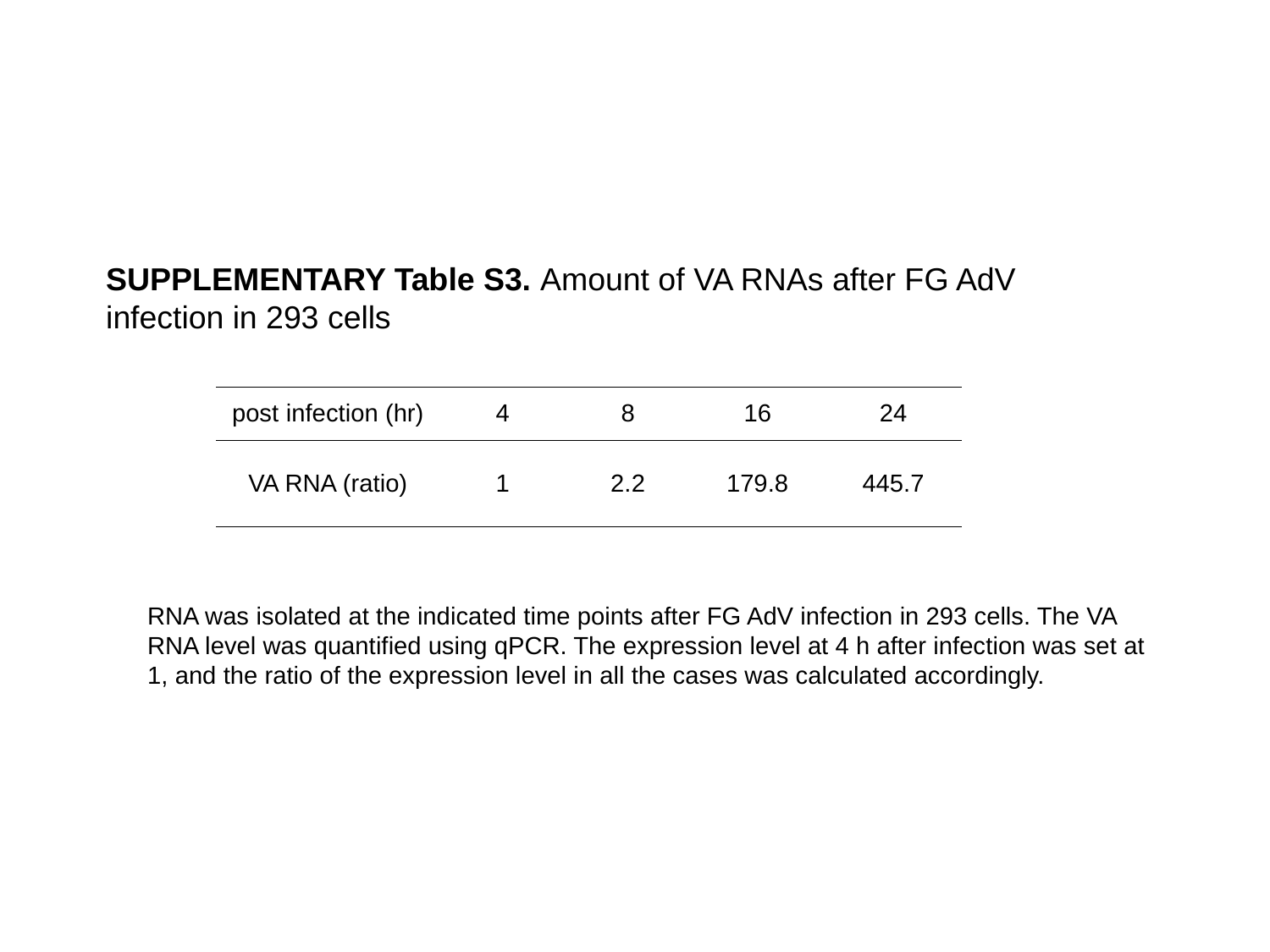

SUPPLEMENTARY Table S3. Amount of VA RNAs after FG AdV infection in 293 cells
| post infection (hr) | 4 | 8 | 16 | 24 |
| --- | --- | --- | --- | --- |
| VA RNA (ratio) | 1 | 2.2 | 179.8 | 445.7 |
RNA was isolated at the indicated time points after FG AdV infection in 293 cells. The VA RNA level was quantified using qPCR. The expression level at 4 h after infection was set at 1, and the ratio of the expression level in all the cases was calculated accordingly.

## Slide 4
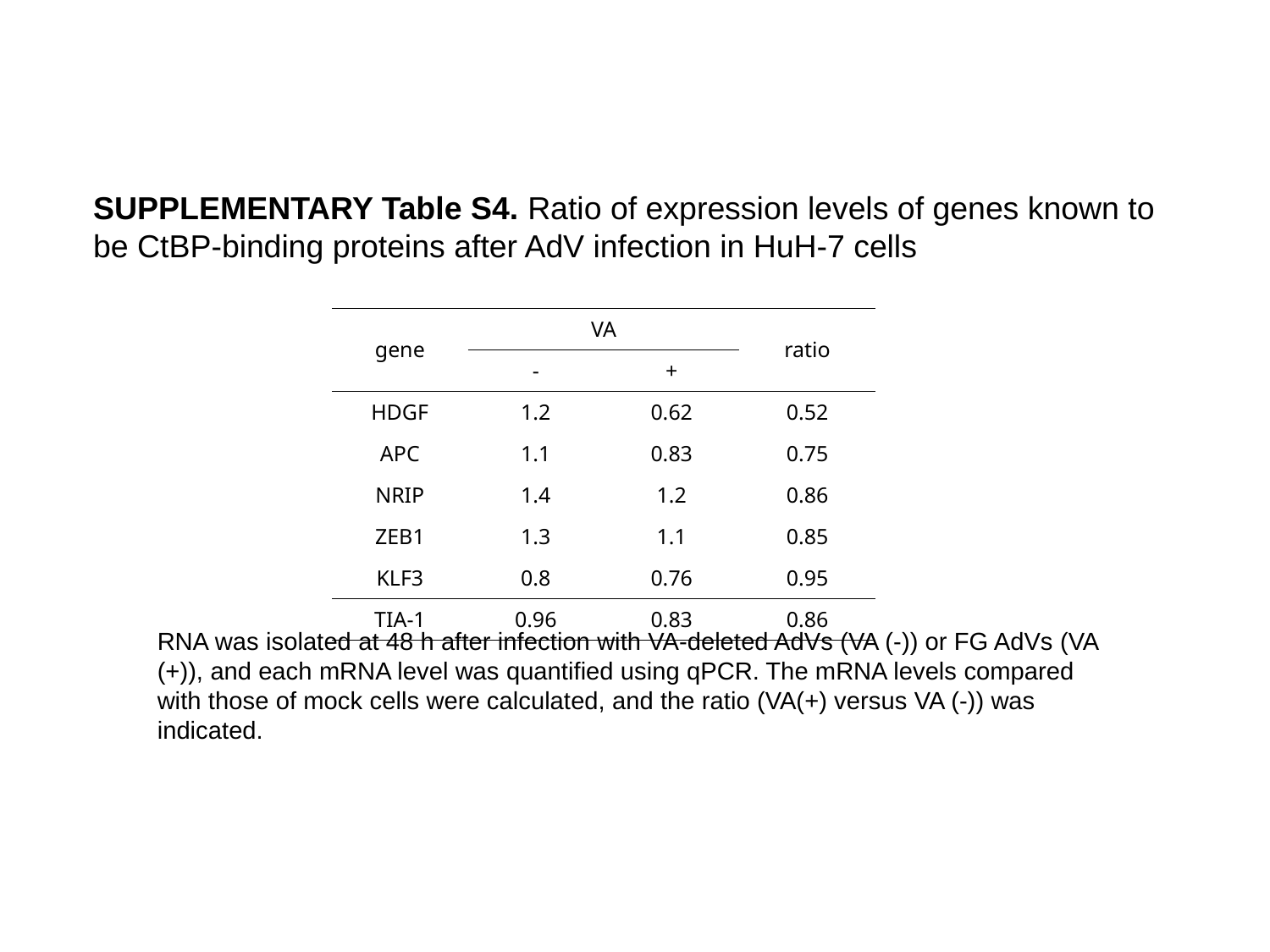

SUPPLEMENTARY Table S4. Ratio of expression levels of genes known to be CtBP-binding proteins after AdV infection in HuH-7 cells
| gene | VA | | ratio |
| --- | --- | --- | --- |
| | - | + | |
| HDGF | 1.2 | 0.62 | 0.52 |
| APC | 1.1 | 0.83 | 0.75 |
| NRIP | 1.4 | 1.2 | 0.86 |
| ZEB1 | 1.3 | 1.1 | 0.85 |
| KLF3 | 0.8 | 0.76 | 0.95 |
| TIA-1 | 0.96 | 0.83 | 0.86 |
RNA was isolated at 48 h after infection with VA-deleted AdVs (VA (-)) or FG AdVs (VA (+)), and each mRNA level was quantified using qPCR. The mRNA levels compared with those of mock cells were calculated, and the ratio (VA(+) versus VA (-)) was indicated.

## Slide 5
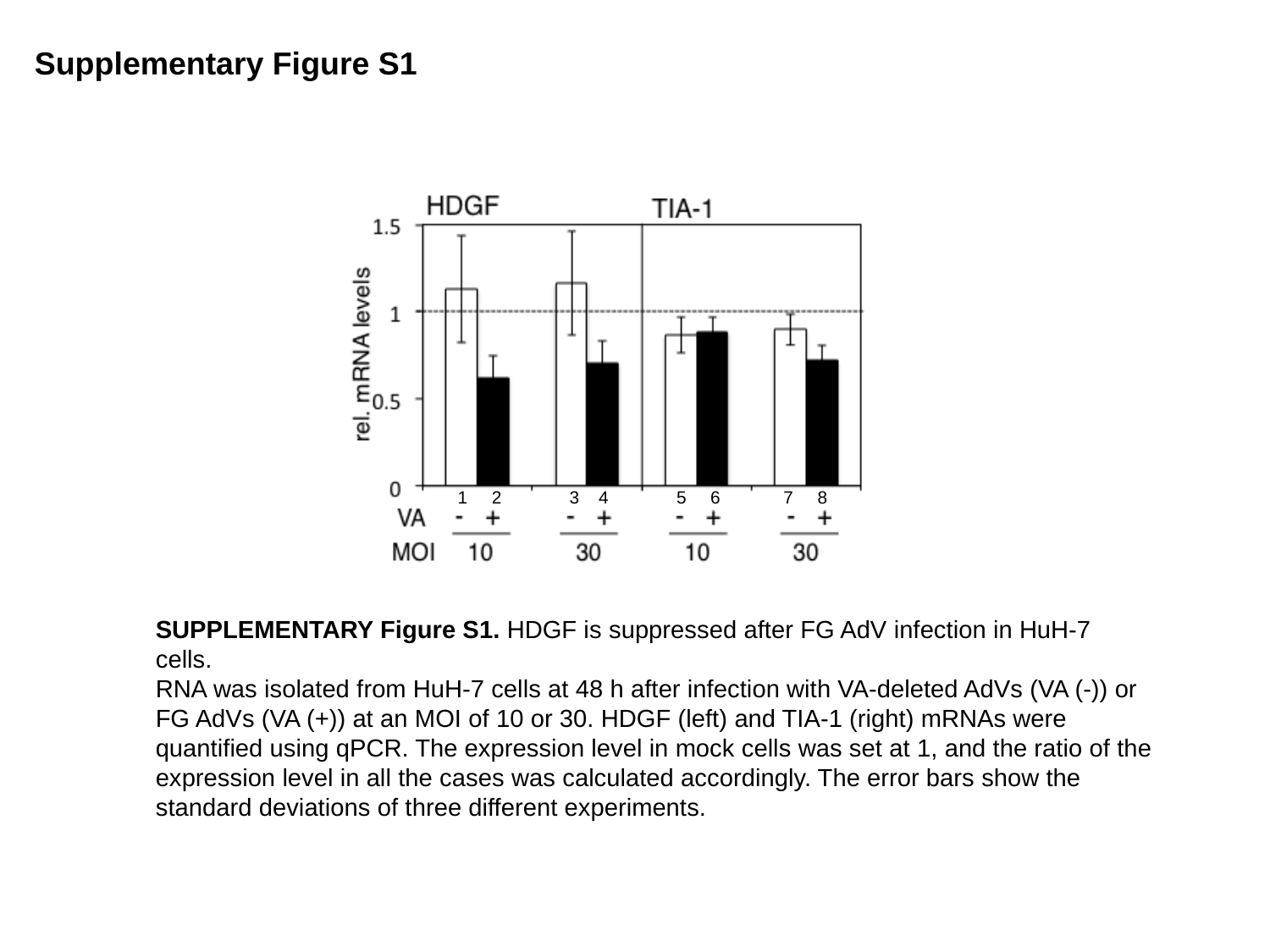

Supplementary Figure S1
1 2 3 4 5 6 7 8
SUPPLEMENTARY Figure S1. HDGF is suppressed after FG AdV infection in HuH-7 cells.
RNA was isolated from HuH-7 cells at 48 h after infection with VA-deleted AdVs (VA (-)) or FG AdVs (VA (+)) at an MOI of 10 or 30. HDGF (left) and TIA-1 (right) mRNAs were quantified using qPCR. The expression level in mock cells was set at 1, and the ratio of the expression level in all the cases was calculated accordingly. The error bars show the standard deviations of three different experiments.

## Slide 6
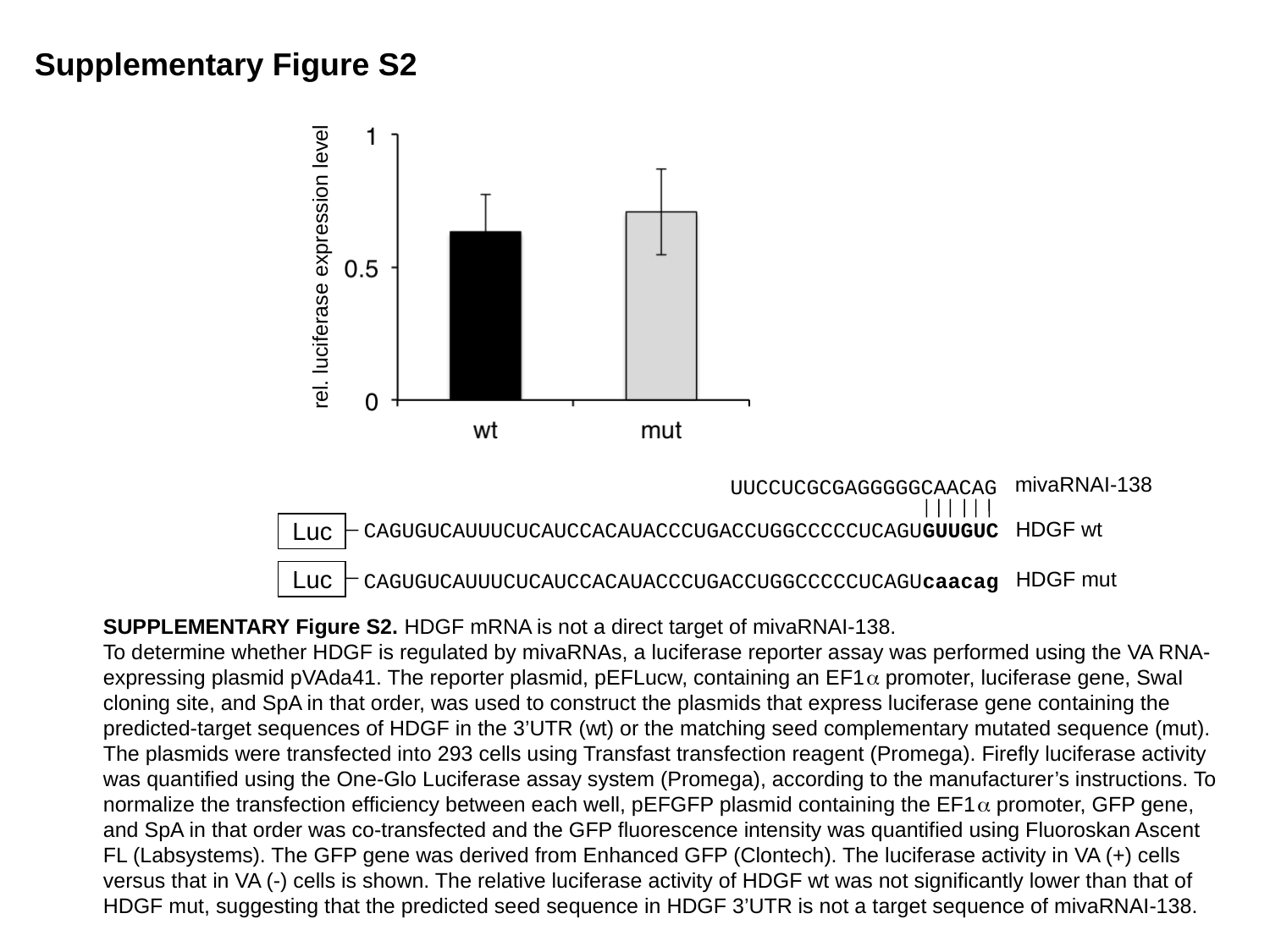

Supplementary Figure S2
rel. luciferase expression level
mivaRNAI-138
UUCCUCGCGAGGGGGCAACAG
HDGF wt
Luc
CAGUGUCAUUUCUCAUCCACAUACCCUGACCUGGCCCCCUCAGUGUUGUC
CAGUGUCAUUUCUCAUCCACAUACCCUGACCUGGCCCCCUCAGUcaacag
Luc
HDGF mut
SUPPLEMENTARY Figure S2. HDGF mRNA is not a direct target of mivaRNAI-138.
To determine whether HDGF is regulated by mivaRNAs, a luciferase reporter assay was performed using the VA RNA-expressing plasmid pVAda41. The reporter plasmid, pEFLucw, containing an EF1α promoter, luciferase gene, SwaI cloning site, and SpA in that order, was used to construct the plasmids that express luciferase gene containing the predicted-target sequences of HDGF in the 3’UTR (wt) or the matching seed complementary mutated sequence (mut). The plasmids were transfected into 293 cells using Transfast transfection reagent (Promega). Firefly luciferase activity was quantified using the One-Glo Luciferase assay system (Promega), according to the manufacturer’s instructions. To normalize the transfection efficiency between each well, pEFGFP plasmid containing the EF1α promoter, GFP gene, and SpA in that order was co-transfected and the GFP fluorescence intensity was quantified using Fluoroskan Ascent FL (Labsystems). The GFP gene was derived from Enhanced GFP (Clontech). The luciferase activity in VA (+) cells versus that in VA (-) cells is shown. The relative luciferase activity of HDGF wt was not significantly lower than that of HDGF mut, suggesting that the predicted seed sequence in HDGF 3’UTR is not a target sequence of mivaRNAI-138.
